# (*Z*)-4-Chloro-*N*-(1-{2-[3-(4-chloro­benzoyl)ureido]eth­yl}imidazolidin-2-yl­idene)benzamide

**DOI:** 10.1107/S1600536812028218

**Published:** 2012-06-27

**Authors:** Dalina Adan, Suhaila Sapari, Siti Nadiah Halim, Bohari M. Yamin

**Affiliations:** a School of Chemical Sciences and Food Technology, Universiti Kebangsaan Malaysia, UKM 43500 Bangi Selangor, Malaysia; b Department of Chemistry, Universiti Malaya, Kuala Lumpur, 50603 Malaysia.

## Abstract

The title compound, C_20_H_19_Cl_2_N_5_O_2_S, was obtained from the reaction of 4-chloro­benzoyl isothio­cyanate with diethyl­ene­triamine. The imidazolidine ring is slightly twisted with an N—C—C—N torsion angle of 15.4 (4)°, while the thio­urea moiety maintains its *trans*–*cis* geometry. The mol­ecule is stabilized by intramolecular N—H⋯O hydrogen bonds. The crystal structure features N—H⋯O, N—H⋯S and C—H⋯O hydrogen bonds and π–π interactions between benzene rings with a centroid–centroid distance of 3.607 (3) Å.

## Related literature

For the structures of bis­(*N*-benzoyl­thio­ureas) derived from aliphatic diamines, see: Ding *et al.* (2008 ▶). For those derived from cyclo­hexane diamine, see: Jumal *et al.* (2011 ▶). For those derived from aromatic diamines, see: Osman & Yamin (2011 ▶); Dong *et al.* (2008 ▶).
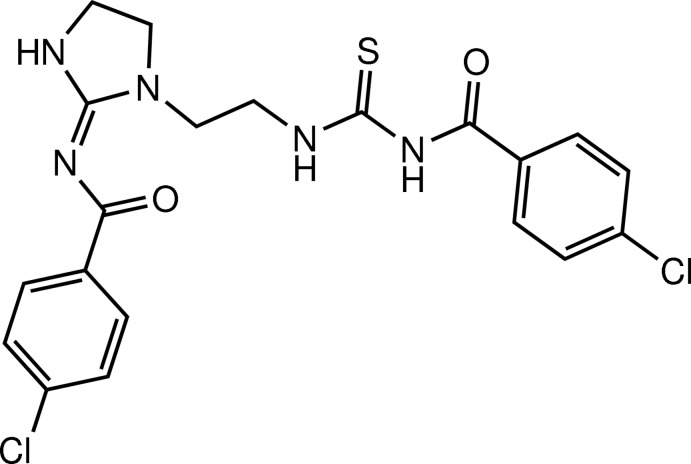


## Experimental

### 

#### Crystal data

C_20_H_19_Cl_2_N_5_O_2_S
*M* *_r_* = 464.36
Monoclinic, 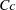

*a* = 24.488 (7) Å
*b* = 6.645 (2) Å
*c* = 13.108 (4) Å
β = 97.16 (2)°
*V* = 2116.2 (11) Å^3^
*Z* = 4
Mo *K*α radiation
μ = 0.43 mm^−1^
*T* = 296 K
0.22 × 0.08 × 0.07 mm

#### Data collection

Bruker SMART APEX CCD area-detector diffractometer
Absorption correction: multi-scan (*SADABS*; Bruker, 2000 ▶) *T*_min_ = 0.911, *T*_max_ = 0.970
8499 measured reflections
4165 independent reflections
2855 reflections with *I* > 2σ(*I*)
*R*_int_ = 0.055

#### Refinement

*R*[*F* ^2^ > 2σ(*F* ^2^)] = 0.040
*wR*(*F* ^2^) = 0.090
*S* = 0.93
4165 reflections
283 parameters
5 restraints
H atoms treated by a mixture of independent and constrained refinement
Δρ_max_ = 0.18 e Å^−3^
Δρ_min_ = −0.28 e Å^−3^
Absolute structure: Flack (1983 ▶) 1987 Friedel pairs
Flack parameter: 0.06 (6)

### 

Data collection: *SMART* (Bruker, 2000 ▶); cell refinement: *SAINT* (Bruker, 2000 ▶); data reduction: *SAINT*; program(s) used to solve structure: *SHELXTL* (Sheldrick, 2008 ▶); program(s) used to refine structure: *SHELXTL*; molecular graphics: *SHELXTL*; software used to prepare material for publication: *SHELXTL*, *PARST* (Nardelli, 1995 ▶) and *PLATON* (Spek, 2009 ▶).

## Supplementary Material

Crystal structure: contains datablock(s) global, I. DOI: 10.1107/S1600536812028218/go2061sup1.cif

Structure factors: contains datablock(s) I. DOI: 10.1107/S1600536812028218/go2061Isup2.hkl

Supplementary material file. DOI: 10.1107/S1600536812028218/go2061Isup3.cml

Additional supplementary materials:  crystallographic information; 3D view; checkCIF report

## Figures and Tables

**Table 1:** Hydrogen-bond geometry (Å, °)

*D*—H⋯*A*	*D*—H	H⋯*A*	*D*⋯*A*	*D*—H⋯*A*
N2—H2*A*⋯O1	0.87 (1)	2.00 (4)	2.637 (4)	130 (4)
N2—H2*A*⋯S1^i^	0.87 (1)	2.85 (4)	3.438 (3)	127 (4)
N4—H4*A*⋯O2	0.86 (1)	1.87 (2)	2.614 (3)	143 (3)
N5—H5*A*⋯O1^ii^	0.86 (1)	2.09 (2)	2.898 (4)	157 (5)
C17—H17*A*⋯O2^iii^	0.93	2.46	3.118 (4)	128

Symmetry codes: (i) 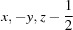; (ii) 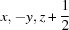; (iii) 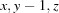.

**Fig. 1.**
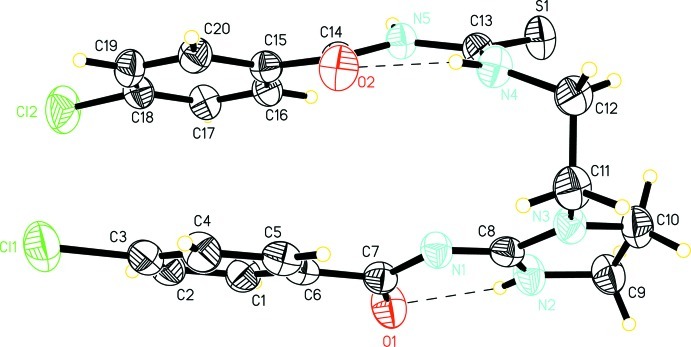
The molecular structure of (I), with displacement ellipsods are drawn at the 50% probability level.

**Fig. 2.**
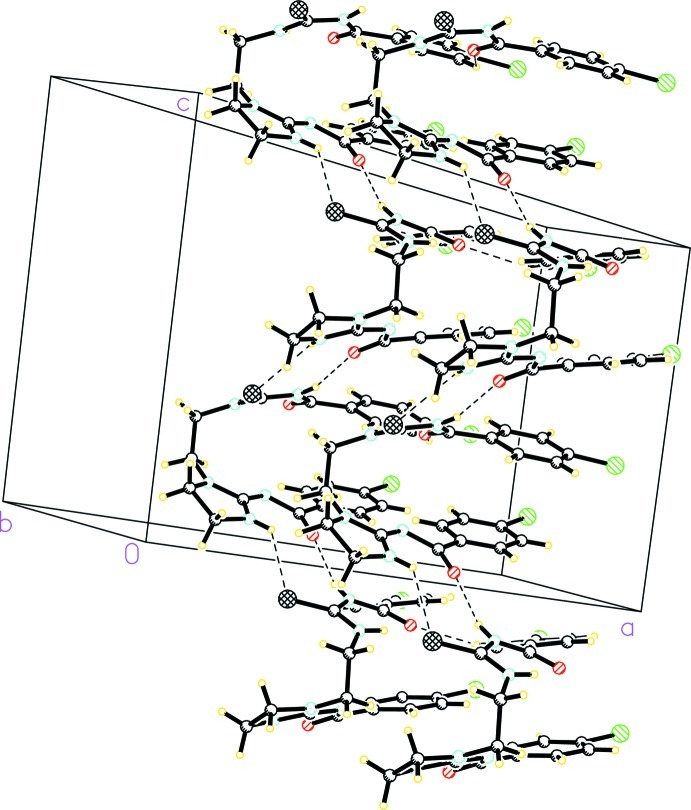
Molecular packing of (I) viewed down b axis. The dashed lines indicate intermolecular hydrogen bonds. Black cross-hatched atoms are S, blue atoms are N and green atoms are Cl.

## supplementary crystallographic information

### Comment

Some bis(thiourea) compounds with several bridges such as 3,3'-dibenzoyl-1,1'-(butane-1,4-diyl)-dithiourea (Ding *et al.*, 2008) 1,2-bis(*N*'-benzoylthioureido)cyclohexane (Jumal *et al.*, 2011) and 1-(4-methylbenzoyl)-3-{2-[3-(4-methylbenzoyl)thioureido]phenyl}thiourea (Osman & Yamin, 2011) have been synthesized by the established reaction between carbonoyl isothiocyanate with the diamines. However,the reaction of 4-chlorobenzoyl isothiocyanate with diethylenetriamine(DIEN) did not give the expected bis or tris(thiourea) but instead, the title compound was obtained indicating a cyclization by the middle nitrogen atom of DIEN to the thiono carbon forming the azomethine funtional group (Fig.1). The molecule consists of the substituted 4-chlorobenzoylimidazolidin-2-ylidene and 4-chlorobenzoylthioureido moiety connected by the ethylene bridge. The N2—C8 and C8—N1 bond lengths of 1.321 (6) and 1.344 (6) Å respectively which are slightly shorter than C8—N3 bond length (1.364 (6) Å) indicates some degree of delocalization along the bonds. The five membered ring N2/N3/C8/C9/C10 is slightly twisted about the C9—C10 bond with these atoms having deviations of of 0.095 (5)Å and -0.091Å from the mean plane of the 5 atoms. The thiourea moiety S1/N4/N5/C13 is planar (maximum deviation 0.003 (3) Å) for atom C13 from the mean plane. The *cis*-*trans* geometry of the thiourea moeity is maintained. The benzene rings of the two hanging arms are nearly co-planar with dihedral angle of 4.88 (19)° between them so forming an intramolecular π–π interaction with a centroid to centroid distance of 3.607 (3)°. There are four intramolecular short contacts, N2—H2A··· O1, N4—H4A···O2,in the molecule. In the crystal sructure, the molecules are linked by N5—H5A···O1(x,-y,1/2+z), N2···H2A···S1(x,-y,-1/2+z) to form a one dimensional zig-zag chain which runs parallel to the *c* axis, Figure 2. These chains are linked and C17—H17A···O2(x,-1+y,z) hydrogen bond forming a two-dimensional sheet which runs parallel to the *bc* plane.

### Experimental

A solution of 4-chlorobenzoylisothiocyanate (1.9162 g, 0.015 mol) in 30 ml acetone was added into a flask containing 20 ml acetone solution of diethylenetriamine (0.543 g, 0.005 mol). The solution mixture was stirred in ice bath for 1 h. The resulting solution was poured onto an ice cubes to yield a yellow precipitate.The precipitated was filtered off, washed with distilled water and left to dried in atmosphere. Good quality crystal were obtained by recrystallization from ethanol.

### Refinement

All H atoms attached to C and N atoms were fixed geometrically and treated as riding with C—H= 0.93 Å(aromatic) or 0.97 Å(methylene)and N—H= 0.86 Å with *U*_iso_(H)=1.2*U*_eq_(C or N). There were 2174 Friedel Pairs.

### Crystal data

**Table:**

C_20_H_19_Cl_2_N_5_O_2_S	*F*(000) = 960
*M**_r_* = 464.36	*D*_x_ = 1.457 Mg m^−^^3^
Monoclinic, *C**c*	Mo *K*α radiation, λ = 0.71073 Å
Hall symbol: C -2yc	Cell parameters from 2230 reflections
*a* = 24.488 (7) Å	θ = 3.1–26.4°
*b* = 6.645 (2) Å	µ = 0.43 mm^−^^1^
*c* = 13.108 (4) Å	*T* = 296 K
β = 97.16 (2)°	Slab, colourless
*V* = 2116.2 (11) Å^3^	0.22 × 0.08 × 0.07 mm
*Z* = 4	

### Data collection

**Table:**

Bruker SMART APEX CCD area-detector diffractometer	4165 independent reflections
Radiation source: fine-focus sealed tube	2855 reflections with *I* > 2σ(*I*)
Graphite monochromator	*R*_int_ = 0.055
Detector resolution: 83.66 pixels mm^-1^	θ_max_ = 26.4°, θ_min_ = 3.1°
ω scan	*h* = −30→30
Absorption correction: multi-scan (*SADABS*; Bruker, 2000)	*k* = −8→8
*T*_min_ = 0.911, *T*_max_ = 0.970	*l* = −16→16
8499 measured reflections	

### Refinement

**Table:**

Refinement on *F*^2^	Secondary atom site location: difference Fourier map
Least-squares matrix: full	Hydrogen site location: inferred from neighbouring sites
*R*[*F*^2^ > 2σ(*F*^2^)] = 0.040	H atoms treated by a mixture of independent and constrained refinement
*wR*(*F*^2^) = 0.090	*w* = 1/[σ^2^(*F*_o_^2^) + (0.0374*P*)^2^] where *P* = (*F*_o_^2^ + 2*F*_c_^2^)/3
*S* = 0.93	(Δ/σ)_max_ < 0.001
4165 reflections	Δρ_max_ = 0.18 e Å^−^^3^
283 parameters	Δρ_min_ = −0.28 e Å^−^^3^
5 restraints	Absolute structure: Flack (1983) ???? Friedel pairs
Primary atom site location: structure-invariant direct methods	Flack parameter: 0.06 (6)

### Special details

**Table:**

Geometry. All esds (except the esd in the dihedral angle between two l.s. planes) are estimated using the full covariance matrix. The cell esds are taken into account individually in the estimation of esds in distances, angles and torsion angles; correlations between esds in cell parameters are only used when they are defined by crystal symmetry. An approximate (isotropic) treatment of cell esds is used for estimating esds involving l.s. planes.
Refinement. Refinement of F^2^ against ALL reflections. The weighted R-factor wR and goodness of fit S are based on F^2^, conventional R-factors R are based on F, with F set to zero for negative F^2^. The threshold expression of F^2^ > 2sigma(F^2^) is used only for calculating R-factors(gt) etc. and is not relevant to the choice of reflections for refinement. R-factors based on F^2^ are statistically about twice as large as those based on F, and R- factors based on ALL data will be even larger.

### Fractional atomic coordinates and isotropic or equivalent isotropic displacement parameters (Å^2^)

**Table:**

	*x*	*y*	*z*	*U*_iso_*/*U*_eq_	
Cl1	0.84274 (4)	0.3989 (2)	0.19364 (9)	0.0858 (4)	
Cl2	0.83019 (4)	−0.17344 (19)	0.37999 (10)	0.0887 (4)	
S1	0.47526 (3)	0.21472 (13)	0.36669 (7)	0.0574 (3)	
O1	0.59035 (9)	0.0679 (4)	0.0292 (2)	0.0654 (7)	
O2	0.63898 (9)	0.5325 (3)	0.35719 (19)	0.0536 (6)	
N1	0.56767 (10)	0.3697 (4)	0.1039 (2)	0.0456 (7)	
N2	0.48754 (11)	0.1718 (5)	0.0424 (2)	0.0564 (8)	
H2A	0.5083 (15)	0.083 (5)	0.018 (3)	0.100 (16)*	
N3	0.47666 (10)	0.4668 (4)	0.1099 (2)	0.0478 (7)	
N4	0.53148 (11)	0.5442 (4)	0.3285 (2)	0.0479 (7)	
H4A	0.5640 (7)	0.584 (5)	0.319 (3)	0.053 (10)*	
N5	0.58290 (10)	0.2711 (4)	0.3890 (2)	0.0426 (6)	
H5A	0.583 (2)	0.153 (3)	0.415 (4)	0.13 (2)*	
C1	0.70080 (13)	0.1240 (6)	0.1077 (3)	0.0509 (9)	
H1B	0.6892	−0.0066	0.0914	0.061*	
C2	0.75617 (14)	0.1603 (6)	0.1344 (3)	0.0550 (9)	
H2B	0.7817	0.0561	0.1368	0.066*	
C3	0.77275 (14)	0.3549 (6)	0.1573 (3)	0.0566 (10)	
C4	0.73592 (13)	0.5075 (5)	0.1551 (3)	0.0534 (9)	
H4B	0.7481	0.6377	0.1710	0.064*	
C5	0.68087 (14)	0.4709 (5)	0.1295 (2)	0.0496 (8)	
H5B	0.6559	0.5764	0.1288	0.060*	
C6	0.66193 (13)	0.2761 (5)	0.1045 (2)	0.0434 (8)	
C7	0.60276 (13)	0.2283 (5)	0.0752 (2)	0.0444 (8)	
C8	0.51449 (13)	0.3336 (5)	0.0849 (2)	0.0433 (8)	
C9	0.42894 (14)	0.2020 (6)	0.0255 (3)	0.0606 (9)	
H9A	0.4095	0.0871	0.0488	0.073*	
H9B	0.4164	0.2259	−0.0466	0.073*	
C10	0.42110 (14)	0.3873 (6)	0.0898 (3)	0.0624 (10)	
H10A	0.3964	0.4832	0.0520	0.075*	
H10B	0.4068	0.3519	0.1532	0.075*	
C11	0.48875 (14)	0.6591 (5)	0.1608 (3)	0.0538 (9)	
H11A	0.4633	0.7594	0.1294	0.065*	
H11B	0.5256	0.7006	0.1504	0.065*	
C12	0.48476 (14)	0.6519 (5)	0.2745 (3)	0.0516 (9)	
H12A	0.4839	0.7878	0.3011	0.062*	
H12B	0.4509	0.5851	0.2862	0.062*	
C13	0.53131 (12)	0.3544 (5)	0.3597 (2)	0.0420 (7)	
C14	0.63297 (12)	0.3540 (5)	0.3762 (2)	0.0412 (7)	
C15	0.68047 (12)	0.2137 (5)	0.3839 (2)	0.0419 (7)	
C16	0.67515 (12)	0.0123 (5)	0.3578 (2)	0.0443 (8)	
H16A	0.6403	−0.0424	0.3402	0.053*	
C17	0.72101 (13)	−0.1086 (5)	0.3576 (3)	0.0483 (8)	
H17A	0.7173	−0.2439	0.3398	0.058*	
C18	0.77199 (13)	−0.0256 (6)	0.3840 (3)	0.0516 (8)	
C19	0.77881 (13)	0.1758 (5)	0.4111 (3)	0.0524 (8)	
H19A	0.8137	0.2295	0.4294	0.063*	
C20	0.73277 (12)	0.2930 (5)	0.4101 (2)	0.0473 (8)	
H20A	0.7366	0.4285	0.4274	0.057*	

### Atomic displacement parameters (Å^2^)

**Table:**

	*U*^11^	*U*^22^	*U*^33^	*U*^12^	*U*^13^	*U*^23^
Cl1	0.0511 (5)	0.1137 (9)	0.0944 (8)	−0.0131 (6)	0.0161 (5)	−0.0142 (7)
Cl2	0.0558 (6)	0.0981 (9)	0.1106 (9)	0.0265 (6)	0.0045 (5)	−0.0239 (8)
S1	0.0419 (4)	0.0488 (5)	0.0824 (7)	0.0017 (4)	0.0109 (4)	0.0106 (5)
O1	0.0544 (13)	0.0527 (15)	0.0894 (19)	−0.0025 (11)	0.0106 (12)	−0.0293 (14)
O2	0.0536 (13)	0.0330 (13)	0.0734 (17)	−0.0069 (10)	0.0045 (11)	0.0035 (12)
N1	0.0427 (15)	0.0405 (16)	0.0538 (18)	0.0054 (12)	0.0067 (13)	−0.0050 (13)
N2	0.0456 (16)	0.0532 (19)	0.071 (2)	0.0027 (14)	0.0082 (14)	−0.0145 (16)
N3	0.0490 (15)	0.0394 (16)	0.0549 (18)	0.0093 (12)	0.0058 (13)	−0.0024 (14)
N4	0.0479 (16)	0.0333 (15)	0.0626 (19)	0.0049 (12)	0.0068 (14)	0.0006 (13)
N5	0.0381 (14)	0.0378 (15)	0.0518 (17)	0.0011 (12)	0.0055 (12)	0.0054 (14)
C1	0.054 (2)	0.050 (2)	0.049 (2)	0.0073 (16)	0.0064 (16)	−0.0063 (17)
C2	0.053 (2)	0.065 (2)	0.049 (2)	0.0126 (17)	0.0112 (16)	−0.0059 (19)
C3	0.050 (2)	0.074 (3)	0.047 (2)	−0.0066 (19)	0.0136 (17)	−0.004 (2)
C4	0.056 (2)	0.049 (2)	0.058 (2)	−0.0078 (17)	0.0134 (17)	−0.0080 (18)
C5	0.059 (2)	0.046 (2)	0.046 (2)	0.0006 (16)	0.0140 (16)	−0.0008 (16)
C6	0.0538 (19)	0.044 (2)	0.0344 (19)	0.0026 (15)	0.0130 (15)	−0.0008 (15)
C7	0.0492 (18)	0.0414 (19)	0.043 (2)	0.0021 (14)	0.0071 (15)	−0.0056 (16)
C8	0.0509 (19)	0.0421 (19)	0.0378 (18)	0.0025 (15)	0.0090 (15)	−0.0011 (15)
C9	0.0486 (19)	0.062 (2)	0.072 (3)	−0.0022 (16)	0.0121 (18)	−0.008 (2)
C10	0.048 (2)	0.068 (3)	0.070 (3)	0.0153 (17)	0.0064 (18)	−0.003 (2)
C11	0.061 (2)	0.0326 (18)	0.068 (3)	0.0122 (15)	0.0082 (18)	0.0048 (17)
C12	0.063 (2)	0.0361 (19)	0.055 (2)	0.0175 (15)	0.0035 (17)	−0.0065 (16)
C13	0.0421 (16)	0.0401 (19)	0.044 (2)	0.0069 (13)	0.0053 (14)	−0.0033 (15)
C14	0.0422 (17)	0.0419 (19)	0.0393 (19)	−0.0029 (14)	0.0041 (13)	−0.0008 (16)
C15	0.0432 (16)	0.0407 (18)	0.0420 (19)	−0.0046 (13)	0.0060 (14)	0.0014 (15)
C16	0.0411 (16)	0.0443 (19)	0.048 (2)	−0.0046 (14)	0.0074 (14)	0.0008 (16)
C17	0.0473 (18)	0.0401 (18)	0.058 (2)	−0.0011 (15)	0.0101 (16)	−0.0010 (17)
C18	0.0441 (17)	0.060 (2)	0.051 (2)	0.0098 (16)	0.0082 (15)	−0.0001 (18)
C19	0.0387 (17)	0.059 (2)	0.058 (2)	−0.0068 (16)	0.0023 (15)	−0.0031 (19)
C20	0.0448 (18)	0.0454 (19)	0.051 (2)	−0.0051 (15)	0.0033 (15)	−0.0003 (16)

### Geometric parameters (Å, º)

**Table:**

Cl1—C3	1.745 (4)	C4—H4B	0.9300
Cl2—C18	1.737 (3)	C5—C6	1.400 (4)
S1—C13	1.669 (3)	C5—H5B	0.9300
O1—C7	1.243 (4)	C6—C7	1.486 (4)
O2—C14	1.224 (3)	C9—C10	1.518 (5)
N1—C8	1.317 (4)	C9—H9A	0.9700
N1—C7	1.358 (4)	C9—H9B	0.9700
N2—C8	1.346 (4)	C10—H10A	0.9700
N2—C9	1.438 (4)	C10—H10B	0.9700
N2—H2A	0.865 (10)	C11—C12	1.505 (5)
N3—C8	1.351 (4)	C11—H11A	0.9700
N3—C10	1.453 (4)	C11—H11B	0.9700
N3—C11	1.455 (4)	C12—H12A	0.9700
N4—C13	1.326 (4)	C12—H12B	0.9700
N4—C12	1.456 (4)	C14—C15	1.484 (4)
N4—H4A	0.864 (10)	C15—C16	1.384 (4)
N5—C14	1.374 (4)	C15—C20	1.388 (4)
N5—C13	1.389 (4)	C16—C17	1.381 (4)
N5—H5A	0.857 (10)	C16—H16A	0.9300
C1—C2	1.378 (5)	C17—C18	1.369 (4)
C1—C6	1.385 (4)	C17—H17A	0.9300
C1—H1B	0.9300	C18—C19	1.389 (5)
C2—C3	1.378 (5)	C19—C20	1.369 (4)
C2—H2B	0.9300	C19—H19A	0.9300
C3—C4	1.355 (5)	C20—H20A	0.9300
C4—C5	1.370 (4)		
			
C8—N1—C7	117.8 (3)	N3—C10—C9	102.4 (3)
C8—N2—C9	112.4 (3)	N3—C10—H10A	111.3
C8—N2—H2A	115 (3)	C9—C10—H10A	111.3
C9—N2—H2A	131 (3)	N3—C10—H10B	111.3
C8—N3—C10	111.9 (3)	C9—C10—H10B	111.3
C8—N3—C11	125.5 (3)	H10A—C10—H10B	109.2
C10—N3—C11	122.3 (3)	N3—C11—C12	113.1 (3)
C13—N4—C12	125.8 (3)	N3—C11—H11A	109.0
C13—N4—H4A	112 (2)	C12—C11—H11A	109.0
C12—N4—H4A	118 (2)	N3—C11—H11B	109.0
C14—N5—C13	127.0 (3)	C12—C11—H11B	109.0
C14—N5—H5A	117 (3)	H11A—C11—H11B	107.8
C13—N5—H5A	116 (3)	N4—C12—C11	110.7 (3)
C2—C1—C6	122.1 (3)	N4—C12—H12A	109.5
C2—C1—H1B	119.0	C11—C12—H12A	109.5
C6—C1—H1B	119.0	N4—C12—H12B	109.5
C3—C2—C1	118.2 (3)	C11—C12—H12B	109.5
C3—C2—H2B	120.9	H12A—C12—H12B	108.1
C1—C2—H2B	120.9	N4—C13—N5	115.3 (3)
C4—C3—C2	121.4 (3)	N4—C13—S1	125.5 (2)
C4—C3—Cl1	120.6 (3)	N5—C13—S1	119.3 (2)
C2—C3—Cl1	118.0 (3)	O2—C14—N5	123.0 (3)
C3—C4—C5	120.3 (3)	O2—C14—C15	120.6 (3)
C3—C4—H4B	119.8	N5—C14—C15	116.3 (3)
C5—C4—H4B	119.8	C16—C15—C20	118.9 (3)
C4—C5—C6	120.6 (3)	C16—C15—C14	122.8 (3)
C4—C5—H5B	119.7	C20—C15—C14	118.0 (3)
C6—C5—H5B	119.7	C17—C16—C15	120.8 (3)
C1—C6—C5	117.4 (3)	C17—C16—H16A	119.6
C1—C6—C7	119.7 (3)	C15—C16—H16A	119.6
C5—C6—C7	122.8 (3)	C18—C17—C16	118.8 (3)
O1—C7—N1	127.1 (3)	C18—C17—H17A	120.6
O1—C7—C6	118.7 (3)	C16—C17—H17A	120.6
N1—C7—C6	114.2 (3)	C17—C18—C19	122.0 (3)
N1—C8—N2	130.2 (3)	C17—C18—Cl2	119.4 (3)
N1—C8—N3	121.8 (3)	C19—C18—Cl2	118.7 (3)
N2—C8—N3	107.9 (3)	C20—C19—C18	118.2 (3)
N2—C9—C10	102.7 (3)	C20—C19—H19A	120.9
N2—C9—H9A	111.2	C18—C19—H19A	120.9
C10—C9—H9A	111.2	C19—C20—C15	121.3 (3)
N2—C9—H9B	111.2	C19—C20—H20A	119.3
C10—C9—H9B	111.2	C15—C20—H20A	119.3
H9A—C9—H9B	109.1		
			
C6—C1—C2—C3	−0.7 (5)	C11—N3—C10—C9	173.4 (3)
C1—C2—C3—C4	0.7 (5)	N2—C9—C10—N3	15.4 (4)
C1—C2—C3—Cl1	178.7 (3)	C8—N3—C11—C12	−99.4 (4)
C2—C3—C4—C5	−0.1 (5)	C10—N3—C11—C12	73.8 (4)
Cl1—C3—C4—C5	−178.0 (3)	C13—N4—C12—C11	−100.0 (4)
C3—C4—C5—C6	−0.6 (5)	N3—C11—C12—N4	73.6 (4)
C2—C1—C6—C5	0.0 (5)	C12—N4—C13—N5	166.5 (3)
C2—C1—C6—C7	180.0 (3)	C12—N4—C13—S1	−14.0 (5)
C4—C5—C6—C1	0.6 (4)	C14—N5—C13—N4	−9.4 (5)
C4—C5—C6—C7	−179.4 (3)	C14—N5—C13—S1	171.1 (3)
C8—N1—C7—O1	2.4 (5)	C13—N5—C14—O2	16.6 (5)
C8—N1—C7—C6	−176.9 (3)	C13—N5—C14—C15	−161.9 (3)
C1—C6—C7—O1	−18.3 (4)	O2—C14—C15—C16	−149.1 (3)
C5—C6—C7—O1	161.6 (3)	N5—C14—C15—C16	29.5 (5)
C1—C6—C7—N1	161.0 (3)	O2—C14—C15—C20	25.3 (5)
C5—C6—C7—N1	−19.0 (4)	N5—C14—C15—C20	−156.2 (3)
C7—N1—C8—N2	1.9 (5)	C20—C15—C16—C17	−0.1 (5)
C7—N1—C8—N3	−179.3 (3)	C14—C15—C16—C17	174.3 (3)
C9—N2—C8—N1	−173.8 (3)	C15—C16—C17—C18	0.1 (5)
C9—N2—C8—N3	7.3 (4)	C16—C17—C18—C19	0.2 (5)
C10—N3—C8—N1	−175.0 (3)	C16—C17—C18—Cl2	−177.9 (2)
C11—N3—C8—N1	−1.2 (5)	C17—C18—C19—C20	−0.6 (5)
C10—N3—C8—N2	4.0 (4)	Cl2—C18—C19—C20	177.5 (3)
C11—N3—C8—N2	177.8 (3)	C18—C19—C20—C15	0.7 (5)
C8—N2—C9—C10	−14.6 (4)	C16—C15—C20—C19	−0.4 (5)
C8—N3—C10—C9	−12.6 (4)	C14—C15—C20—C19	−174.9 (3)

### Hydrogen-bond geometry (Å, º)

**Table:**

*D*—H···*A*	*D*—H	H···*A*	*D*···*A*	*D*—H···*A*
N2—H2*A*···O1	0.87 (1)	2.00 (4)	2.637 (4)	130 (4)
N2—H2*A*···S1^i^	0.87 (1)	2.85 (4)	3.438 (3)	127 (4)
N4—H4*A*···O2	0.86 (1)	1.87 (2)	2.614 (3)	143 (3)
N5—H5*A*···O1^ii^	0.86 (1)	2.09 (2)	2.898 (4)	157 (5)
C17—H17*A*···O2^iii^	0.93	2.46	3.118 (4)	128

Symmetry codes: (i) *x*, −*y*, *z*−1/2; (ii) *x*, −*y*, *z*+1/2; (iii) *x*, *y*−1, *z*.
